# Authors’ response to the invited commentary on decellularized aortic allografts for paediatric aortic valve replacement

**DOI:** 10.1093/ejcts/ezae212

**Published:** 2024-06-05

**Authors:** Alexander Horke, Dietmar Boethig, Samir Sarikouch

**Affiliations:** Department for Cardiothoracic, Transplant, and Vascular Surgery, Hannover Medical School, Germany; Department for Cardiothoracic, Transplant, and Vascular Surgery, Hannover Medical School, Germany; Department for Cardiothoracic, Transplant, and Vascular Surgery, Hannover Medical School, Germany

**Keywords:** Children, Aortic valve disease, Decellularization, Allografts

We read with great interest the invited commentary by Dr Bove [1] on the recent publication of our 5-year follow-up study on decellularized aortic homografts for paediatric aortic valve replacement [2]. While we acknowledge and fully agree with the majority of the issues discussed in the above, e.g. that decellularized aortic homografts have not met all initial expectations based on findings suggesting a low-dose immune response leading to reoperation, we feel it is important to provide the correct context for the points raised.

In our view, the critique regarding a lower rate of freedom from reoperation in comparison to the results of paediatric Ross operations needs to be considered in light of the specific demographic of the study cohort. The Paediatric Subgroup of the prospective ARISE trial is characterized by a cohort composition with the largest number of previous cardiac surgeries and, in particular, a significantly higher rate of previous aortic valve replacements compared with other cohorts. 84/143 children (59%) had undergone previous cardiac operations (47 children with 1, 24 with 2, 13 with ≥3 previous cardiac operations). Twenty-four (17%) had undergone previous aortic valve replacements (19 children with 1, 4 with 2 and 1 with 3).

Ross patients within the STS Congenital Heart Surgery Database underwent surgical cardiac procedures only in 43.5% [3]. In a recent review on the results of paediatric aortic valve replacement by Notenboom *et al.* [4] published in the *European Heart Journal* the percentage of previous surgical cardiac procedures was even less with 29.1% in children treated with the Ross operation and the number of previous aortic valve replacements only 1.8%. A dedicated Ross review by Etnel *et al.* [5] analysing >2700 children did report previous cardiac surgery in 32.3% and previous aortic valve replacements only in 2.3% of the patients. The only paediatric cohort with a similar prevalence of previous surgery stems from Quebec, where the colleagues had 53.9% previous cardiac operations, but again the rate for previous aortic valve replacement was significantly less with 4.7% [6].

We view decellularized aortic homografts as an additional therapeutic avenue for children, who require an aortic root procedure, but who are unsuitable candidates for a durable Ross operation. They also offer an alternative for children with contraindications for mechanical aortic valve replacement. In the future, subcoronary implantation of decellularized aortic homografts may provide an option to avoid root replacement procedures and coronary transfers in these patients.

**Conflict of interest:** Corlife oHG, the company providing the patented service of processing decellularized allografts, is a spin-off company of the Hannover Medical School. The 3 authors declare that no competing interests exist.

## References

[1] Bove T. Decellularized aortic allografts for aortic valve replacement in children: a valid option? Eur J Cardiothorac Surg 2024;65:ezae145. 10.1093/ejcts/ezae145 38579271

[2] Horke A , Bobylev D, Avsar M, Cvitkovic T, Meyns B, Rega F et al Paediatric aortic valve replacement using decellularized allografts: a multicentre update following 143 implantations and five-year mean follow-up. Eur J Cardiothorac Surg 2024;65:ezae112. 10.1093/ejcts/ezae112 PMC11001491 38532286

[3] Rowe G , Gill G, Zubair MM, Roach A, Egorova N, Emerson D et al Ross procedure in children: the Society of Thoracic Surgeons Congenital Heart Surgery Database Analysis. Ann Thorac Surg 2023;115:119–25. 35870519 10.1016/j.athoracsur.2022.06.043

[4] Notenboom ML , Schuermans A, Etnel JRG, Veen KM, van de Woestijne PC, Rega FR et al Paediatric aortic valve replacement: a meta-analysis and microsimulation study. Eur Heart J 2023;44:3231–46. 37366156 10.1093/eurheartj/ehad370 PMC10482570

[5] Etnel JRG , Grashuis P, Huygens SA, Pekbay B, Papageorgiou G, Helbing WA et al The Ross procedure: a systematic review, meta-analysis, and microsimulation. Circ Cardiovasc Qual Outcomes 2018;11:e004748. 30562065 10.1161/CIRCOUTCOMES.118.004748

[6] Martin E , Laurin C, Jacques F, Houde C, Cote JM, Chetaille P et al More than 25 years of experience with the Ross procedure in children: a single-center experience. Ann Thorac Surg 2020;110:638–44. 31881194 10.1016/j.athoracsur.2019.10.093

